# Occurrence of Secondary Non-Hodgkin Lymphomas Among Our Classical Hodgkin Lymphoma Patients: A Single-Centre Experience

**DOI:** 10.7759/cureus.63307

**Published:** 2024-06-27

**Authors:** Bálint Virga, László Pinczés, Árpád Illés, Zsófia Miltényi, Ferenc Magyari, Gábor Méhes, Zsófia Simon

**Affiliations:** 1 Internal Medicine, University of Debrecen, Debrecen, HUN; 2 Clinical Medicine, University of Debrecen, Debrecen, HUN; 3 Pathology, University of Debrecen, Debrecen, HUN

**Keywords:** personalized therapy, aggressive treatment, synchronous tumors, non-hodgkin lymphoma, classical hodgkin lymphoma, secondary malignancies

## Abstract

Objective

Non-Hodgkin lymphoma (NHL) arising as a secondary malignancy in patients treated for classical Hodgkin lymphoma (cHL) is an infrequent and challenging clinical scenario. NHL can be presented synchronously with cHL or may develop later, sequentially, up to years after treatment for cHL. The relationship between the two lymphomas is unclear, and there are no clear guidelines for the management of these patients. We would like to find a better clinical understanding of this issue so this study investigates the occurrence and clinical characteristics of secondary NHL.

Materials and methods

In this retrospective cohort examination, we collected cHL cases when NHL occurred during or after the course of treating cHL. We performed the histopathologic revisions of the samples, and in every case where the quality of the sample was lower, we performed molecular examinations to find the association between cHL and NHL. We performed next-generation genome sequencing (NGS) and immunoglobulin heavy-chain variable region gene (IgHV) clonality testing.

Results

In a cohort of 164 cHL patients diagnosed between 2011 and 2020, six patients were identified with NHL during rebiopsy prompted by lymphoma relapse or progression. Among these, five patients were diagnosed with post-germinal center-originated diffuse large B-cell lymphoma (post-GC DLBCL), and one patient presented high-grade B-cell lymphoma (HG-BCL). The NHL manifestation differed in its timing: three cases emerged after successful cHL treatment, with at least 18 months of complete remission, while the other three patients faced primary refractory cHL. Notably, the primary refractory cases did not exhibit a confirmed clonal relationship between cHL and NHL, but NGS data raised the possibility of synchronous NHL in one case. In contrast, among the patients with sequentially occurring NHL, polymerase chain reaction (PCR) testing of the IgHV gene affirmed a clonal connection between cHL and secondary DLBCL in one case, while the high morphological similarity suggested a potential clonality between the two lymphomas in another case.

Conclusion

This study reveals that secondary NHL may manifest both synchronously and sequentially following cHL. Our results suggest that synchronous NHL has a worse prognosis compared to sequential cases when the different lymphomas are not recognized at the time of diagnosis. As our data showed, in some cases, mutations that accompany the tumor cells throughout their clonal evolution can be identified, with additional mutations later on. In the future, next-generation sequencing (NGS)-based processing of liquid biopsy samples can overcome the limitations resulting from the spatial heterogeneity of lymphoid malignancies. Over the long term, this identification could lead to early patient selection and alternative treatment strategies, ultimately leading to improved prospects for cure.

## Introduction

Hodgkin lymphoma (HL) is a lymphoid neoplasm of B-cell origin, most commonly observed in young adults but can occur at any age. It has emerged as a notable success in the field of oncohematology, with the majority of patients achieving a cure. However, a subset of 10 to 15% of HL patients face a grim prognosis, characterized by primary refractory or relapsed disease, which presents significant challenges for clinicians [1].

While the long-term follow-up of HL survivors may unveil treatment-related side effects, investigating the factors contributing to treatment resistance in suboptimal responders holds the potential to enhance our understanding of treatment failures. This knowledge may, in turn, facilitate the development of more personalized and effective treatment strategies [1].

According to the WHO classifications introduced in 2001, HL is categorized into two primary subtypes [2]. The more prevalent form, classical Hodgkin lymphoma (cHL), accounts for the vast majority of cases (95-97%), while the remaining 3-5% of patients are diagnosed with nodular lymphocyte-predominant Hodgkin lymphoma (NLPHL).

The distinction between these two subtypes is rooted in their differing pathological characteristics and clinical behavior. NLPHL exhibits close pathological ties to T-cell/histiocyte-rich large B-cell lymphoma and, clinically, bears a greater resemblance to indolent B-cell lymphomas. This resemblance is evident in its favorable response to treatment, the tendency for late relapses, and a somewhat higher likelihood (3-7%) of transformation into non-Hodgkin lymphoma (NHL). In light of these considerations, the International Consensus Classification (ICC) has proposed new terminology for the disease, referring to it as nodular lymphocyte-predominant B-cell lymphoma [3].

In contrast, the transformation of cHL into NHL is a rare occurrence, transpiring in only 1-2% of cases. Notably, both types of lymphoid malignancies, cHL and NHL, can coexist within the same lymph node, leading to a condition known as composite lymphoma. Additionally, they can manifest concurrently but in different anatomical locations, referred to as synchronous or discordant lymphoma. However, the occurrence of these phenomena is infrequent and often goes underrecognized. The pathogenesis of composite and synchronous lymphomas can be attributed to the discordant differentiation of neoplastic cells with a common origin. Typically, patients diagnosed with composite or synchronous lymphoma exhibit a favorable response to initial immuno-chemotherapy. More frequently, NHL is identified as a secondary neoplasm following the initial treatment of cHL when a rebiopsy is conducted due to suspected relapse or refractoriness of the disease. It is well-established that among cHL patients, the risk of developing secondary malignancies is elevated. This profound risk can be attributed to the side effects of chemotherapy and radiotherapy, immunoregulatory disturbances induced by cHL itself, or treatment-related immunodeficiency [4].

It is important to acknowledge the existence of grey zone lymphoma (GZL), a clinical entity that lies in between cHL and diffuse large B-cell lymphoma (DLBCL). GZL represents a distinct, albeit rare, category and must be differentiated from secondary NHL cases [5,6]. In the current context, our deliberate focus is on the occurrence of secondary NHL, and we are excluding intermediate lymphomas from our discussion.

The diagnosis and treatment of secondary NHLs present notable challenges, compounded by a scarcity of literature on the topic. These patients face a dismal, with frequent treatment failures despite diverse therapeutic strategies [7-9]. Our objective is to assemble cases of cHL patients in whom NHL developed subsequent to the initial cHL diagnosis. Through this compilation, we aim to present the clinical and pathological characteristics, treatment approaches, and molecular findings of these patients. Our overarching goal is to identify common underlying factors in the development of this co-occurrence, enhancing our understanding of the pathophysiology of these conditions.

## Materials and methods

We conducted a retrospective data review of cHL patients who received treatment at the Department of Hematology of the University of Debrecen, between January 2011 and December 2020. Data were retrospectively extracted from electronic medical records. Given the retrospective nature of our study, specific informed consent for the study itself was not required. Nevertheless, all procedures, examinations, and interventions were carried out following the patients' informed consent.

We systematically identified cases in which NHL was diagnosed subsequent to cHL, and we collected their clinical and pathological data. A highly experienced hematopathologist meticulously reviewed all available tissue blocks, confirming the diagnoses of both cHL and NHL. Clonality examinations were conducted in the Department of Pathology, University of Debrecen whenever sufficient tissue samples were obtainable. However, in some instances, DNA isolation was not feasible, resulting in incomplete clonality test results.

Reverse transcription-polymerase chain reaction (PCR) was performed using the Advantage-2 PCR kit (Clontech, Mountain View, CA, USA). For next-generation sequencing (NGS), we utilized the Archer Fusion Plex Lymphoma panel (Archer, Boulder, CO, USA), a targeted NGS product designed to concurrently detect and identify fusions, point mutations, and expression levels in 125 genes associated with lymphoma. This was accomplished using Anchored Multiplex PCR (AMP)-based enrichment. NGS procedures were conducted on genomic RNA extracted from representative formaldehyde-fixed and paraffin-embedded samples, employing the Illumina platform (Albany, NY, USA). The sequence analysis was performed using the Archer analysis tool. Furthermore, immunoglobulin heavy-chain variable region gene (IgHV) clonality testing was executed on three VH regions (FR1, FR2, and FR3) following the standard Biomed II methodology.

## Results

Between 2011 and 2020, a total of 164 cHL patients received treatment at the Department of Hematology, University of Debrecen. Among them, six individuals (3.65%) were identified as having developed secondary NHL. Among these cases, one patient was diagnosed with high-grade B-cell lymphoma (HG-BCL), while the remaining five exhibited post-germinal center (post-GC) DLBCL. Notably, the occurrence of secondary NHL followed at least 18 months of complete remission (CR) from cHL in three cases, whereas the other three patients had primary refractory disease.

Given the limited number of patients and the variability in disease progression, we opt for presenting each case individually. Subsequently, we provide a summary of the results of the clonality examinations. A comprehensive overview of the clinical data and molecular findings is presented in Table 1.

**Table 1:** Clinical characteristics and molecular findings in our case series. cHL, classical Hodgkin’s lymphoma; DLBCL, diffuse large B-cell lymphoma; NHL, non-Hodgkin lymphoma; NA, not applicable; ND, not done; CMR, complete metabolic remission; ABVD, doxorubicin, bleomycin, vinblastine, dacarbazine; BV, brentuximab vedotin; BEAM: carmustine, etoposide, cytosine arabinoside, melphalan; AHSCT, autologous hematopoietic stem cell transplantation; IFRT, involved field irradiation; R, rituximab; B, bendamustine; ICE, Ifosfamide, carboplatin, etoposide; CHOP, cyclophosphamide, doxorubicin, vincristine, prednisolone; PRB, polatuzumab vedotin, rituximab, bendamustine

Case	Diagnosis (in order of sampling)	1st line treatment	Type of disease progression (time to relapse, month)	Further treatments	Disease status	Survival (months)	Sample’s quality	NGS gene variant
Case #1 (20y F)	Follicular hyperplasia	ABVD	Primary refractory	R-DHAP, BV-ICE, BV-BEAM+ AHSCT Nivolumab IFRT	Dead	OS: 19	Good	NOTCH2, CCND3
	cHL, NS						NA	ND
	HG-BCL					Post-NHL: 2	Good	NOTCH2, PIM1, CIITA, LMO2, CCND3, BATF3, PLCG2
Case #2 (46y M)	cHL, NS	ABVD	Primary refractory	DHAP, BV-B, R-ICE, Nivolumab	Dead	OS: 13	Good	No alterations
	DLBCL					Post-NHL: 4	Good	CCND3, PTPN1, STAT6
Case #3 (42y M)	cHL, NS	ABVD	Primary refractory	DHAP, BV-Benda, BV-BEAM+AHSCT, IFRT, R-B	Dead	OS: 28	Good	ND
	DLBCL					Post-NHL: 2	NA	ND
Case #4 (48y F)	cHL, NS	ABVD	CMR (23)	DHAP, BV-BEAM, AHSCT, R-B, R-CHOP	Dead	OS: 60	NA	ND, poor sample quality
	DLBCL					Post-NHL: 12	NA	ND
Case #5 (68y F)	cHL, MC	EBVD	CMR (18)	Liver resection	Alive	OS: 66	Good	No alterations
	DLBCL					Post-NHL: 27	Good	DEK
Case #6 (32y F)	cHL, NS	ABVD	CMR (52)	R-ICE, BV-DHAP, BV-BEAM, AHSCT, BV, IFRT, PRB	Dead	OS: 72	Good	No alterations
	DLBCL					Post-NHL:15	Good	STAT6, BATF3

Primary refractory patients

Case #1

A cervical lymph node biopsy initially indicated follicular hyperplasia in a 20-year-old woman. However, several months later, a subsequent biopsy revealed a diagnosis of a nodular sclerosis subtype cHL. A staging positron emission tomography and computed tomography (PET/CT) scan demonstrated stage III disease with a notably high maximal standard uptake value (SUVmax) of 25.2, primarily due to a bulky mediastinal tumor.

Following two cycles of standard ABVD (adriablastine, bleomycin, vinblastine, dacarbazine) therapy, an interim PET/CT scan revealed a partial response (PR). Regrettably, the restaging examination showed progression in the supradiaphragmal region. Subsequently, the patient was referred to our center with the intention of undergoing autologous hematopoietic stem cell transplantation (AHSCT) for primary refractory disease. Although there was consideration of a high-grade transformation of the cHL, a mediastinal biopsy was deemed infeasible due to the patient's overall condition and the peak of the coronavirus disease 2019 (COVID-19) pandemic.

We initiated salvage therapy with the DHAP regimen (dexamethasone, cytarabine, cisplatin), supplemented by rituximab, which unfortunately proved ineffective. We then switched to the brentuximab vedotin (BV)+ICE regimen (ifosfamide, carboplatin, etoposide), resulting in a PR. Subsequently, the patient received a high-dose chemotherapy regimen with BV+BEAM (carmustine, etoposide, cytosine arabinoside, melphalan) conditioning, followed by AHSCT. On post-transplant day 77, while the patient was undergoing post-transplant maintenance with BV therapy, a palpable lesion emerged on the chest wall. Histological analysis confirmed the presence of HG-BCL with a distinct immunophenotype.

**Figure 1:**
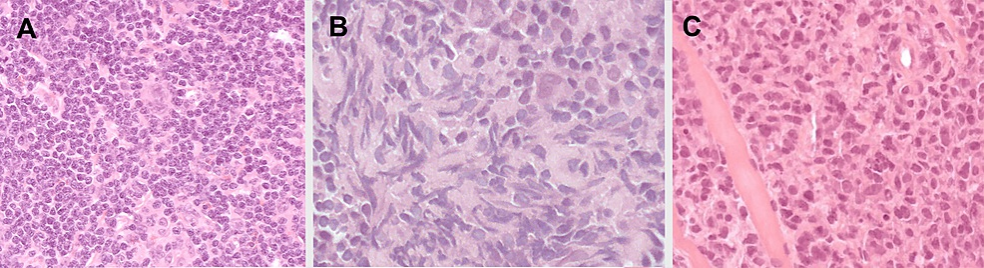
Case #1: 20-year-old female. A: First sample, lymph node biopsy, HE stain, diagnosed to follicular hyperplasia, later revised to cHL. B: Second sample, cervical lymph node biopsy, HE stain, diagnosed to cHL. C: Third sample, mediastinal lymph node biopsy, HE stain: diffuse lymphoid infiltration. cHL, classical Hodgkin lymphoma; HE, hematoxylin-eosin

Following radiation therapy for the mediastinal tumor, nivolumab therapy was initiated. Unfortunately, the patient developed severe renal failure due to underlying lymphomatous infiltration of the kidneys. Subsequent active therapy became unfeasible when the patient contracted a COVID-19 infection, ultimately leading to her passing. The overall survival was 19 months from the diagnosis of cHL and two months from the diagnosis of HG-BCL. Upon histological revision conducted for scientific purposes, the initial biopsy specimen, previously deemed as follicular hyperplasia, was found to contain Hodgkin-Reed-Sternberg (HRS) cells. The histological patterns of the patient are shown in Figure 1.

Case #2

A 46-year-old man received a diagnosis of nodular sclerosing subtype of cHL. A staging PET/CT scan revealed stage III disease. The SUVmax of the mediastinal mass was 22.5, without evidence of bulky disease.

After two cycles of ABVD therapy, complete metabolic remission was observed on the interim PET/CT, with a Deauville Score (DS) of 3. Consequently, the patient continued with the ABVD treatment, although he chose to decline the sixth cycle of therapy. However, within two months of completing the last course of ABVD, the patient presented with symptoms indicative of impending superior vena cava syndrome (SVCS). The clinical symptoms resulted from the significant progression of a mediastinal mass. Despite initiating rescue DHAP therapy, SVCS progressed, necessitating urgent mediastinal irradiation, concomitant with BV+bendamustine therapy.

While his symptoms improved following treatment, a new lymph node appeared in the right cervical region. A histological biopsy confirmed the diagnosis of post-GC DLBCL. Unfortunately, further progression was observed even after R-ICE and nivolumab treatments, ultimately leading to the patient's passing. The overall survival was 13 months from the diagnosis of cHL, with survival after the diagnosis of NHL extending to four months.

Case #3

A 42-year-old man diagnosed with the nodular sclerosing subtype of cHL exhibited disseminated disease (stage IV) on the staging PET/CT scan. Notably, a mediastinal bulky tumor with a significantly elevated SUVmax of 15.5 was observed. We initiated ABVD treatment, which resulted in a PR (DS: 4) on the interim PET/CT scan after the second cycle. Further improvement (DS: 2) was noted following the completion of the fourth cycle.

However, a restaging PET/CT scan, performed after six cycles of ABVD, revealed the appearance of a new mediastinal lesion with a DS of 5. Consequently, the patient was referred to our clinic for consideration of AHSCT. Although salvage DHAP therapy proved ineffective, a transition to BV+bendamustine therapy resulted in a favorable PR, enabling the patient to undergo transplantation.

On the post-transplant PET/CT scan, the mediastinal tumor exhibited progression, with a substantial increase in SUVmax to 20.1. Subsequent treatments, including mediastinal irradiation and nivolumab therapy, failed to yield positive outcomes, and the patient's condition continued to deteriorate. A transbronchial sampling confirmed the diagnosis of post-GC DLBCL. Then he received palliative care, and succumbed to progressive disease 28 months after the initial diagnosis of cHL and just two months after the diagnosis of NHL.

Relapsed patients

Case #4

A 48-year-old woman was diagnosed with the nodular sclerosis histological subtype of cHL at stage III and was initiated on standard ABVD therapy. However, after the second cycle of ABVD, a progressive disease with a DS of 5 was identified on the PET/CT scan. Consequently, she began salvage DHAP therapy, but her disease proved to be refractory to chemotherapy, rendering her ineligible for AHSCT. Subsequently, she was enrolled in a clinical trial and received pembrolizumab therapy. Unfortunately, after the fifth cycle of treatment, her disease progressed, leading to withdrawal from the trial. She was then referred to our center.

With subsequent BV+bendamustine therapy, she achieved CR and underwent AHSCT. Following a post-transplantation maintenance regimen of BV therapy, a post-transplant PET/CT scan indicated disease progression. We transitioned the patient to nivolumab therapy. She received a total of 32 cycles of nivolumab and remained in CR for 19 months.

**Figure 2:**
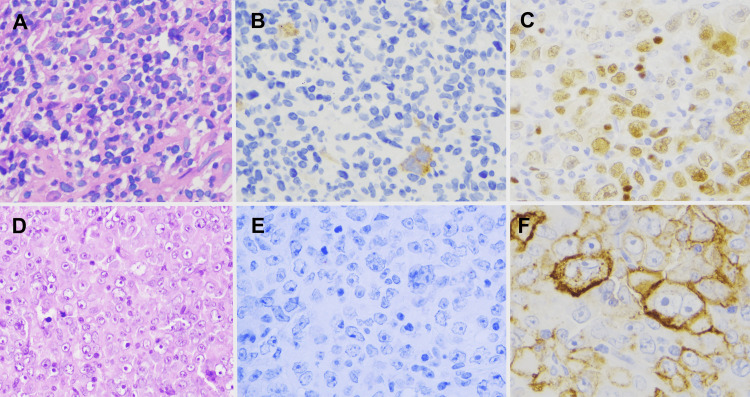
Case#4: 48-year-old female. A, B: Cervical lymph node biopsy, diagnosed to cHL. A: Hematoxylin-eosin stain; B: CD30 immunofluorescent stain. C, D, E, F: A supraclavicular lymph node biopsy diagnosed as DLBCL. D: Hematoxylin-eosin stain; E: CD30 immunofluorescent stain; F: CD20 immunofluorescent stain; C: PAX5 immunofluorescent stain. DLBCL, diffuse large B-cell lymphoma; cHL, classical Hodgkin Lymphoma

However, after this period, a left supraclavicular lymph node became palpable, and histological examination confirmed a transformation of cHL into a post-GC DLBCL. Subsequent therapies involving rituximab+bendamustine and R-CHOP (rituximab, cyclophosphamide, vincristine, prednisolone) were administered. Unfortunately, these treatments were ineffective and tolerated poorly by the patient. With progressive disease, the patient died of COVID-19 infection, after an OS of 69 months from the diagnosis of cHL and 12 months after the diagnosis of NHL. The interval between the diagnosis of cHL and NHL was 57 months. The histological patterns of the patient are shown in Figure 2. 

Case #5

A 68-year-old woman presented with a diagnosis of mixed cellularity subtype of cHL, and a staging PET/CT scan revealed disseminated disease (stage IV), characterized by nodular infiltration of the liver and spleen. Given her cardiac comorbidities, she received a total of six cycles of EBVD (epirubicin, bleomycin, vinblastine, dacarbazine) therapy, which led to a CR.

However, 18 months after first-line therapy, a routine follow-up abdominal ultrasound examination identified a focal hepatic lesion, raising concerns of a potential relapse or a secondary malignancy. The oncology team recommended the resection of the affected liver lobe. From the surgical sample, the histopathological examination confirmed the presence of post-GC DLBCL. It is noteworthy that no signs of nodal or extranodal involvement were evident beyond the resected hepatic lobe.

**Figure 3:**
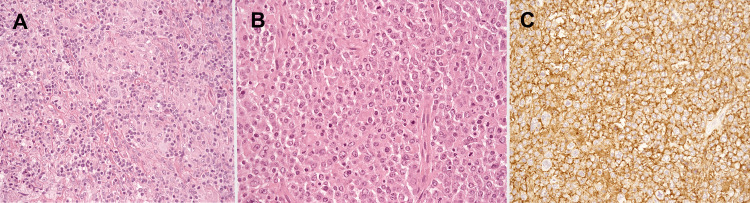
Case #5: 68-year-old female. A: Cervical lymph node biopsy with Hematoxylin-eosin stain diagnosed to cHL. B, C: Sample of a hepatic resection diagnosed to DLBCL. B: Hematoxylin-eosin stain. C: CD20 immunofluorescent stain. cHL, classical Hodgkin lymphoma; DLBCL, diffuse large B-cell lymphoma

Given the localized nature of the recurrence and the absence of other disease manifestations, we adopted a strategy of watchful waiting, and no further treatment was administered. Remarkably, the patient still remains in sustained remission. By the time of the data cut-off date of this study, her overall survival extended to 85 months, with post-NHL overall survival spanning 67 months. The time interval between the diagnosis of cHL and NHL was 18 months. The histological patterns of the patient are shown in Figure 3. 

Case #6

A 32-year-old woman was diagnosed with the nodular sclerosing subtype of cHL. The staging PET/CT scan revealed supradiaphragmatic nodal involvement and nodular pulmonary lesions. She underwent six cycles of ABVD therapy and achieved CR, which lasted for almost five years.

However, a chest CT scan detected a mediastinal tumor mass, raising suspicion of a relapse of cHL. Thoracoscopic sampling and subsequent histological examination confirmed the diagnosis of post-GC DLBCL. At this point, she was referred to our center with the intention of undergoing AHSCT.

After a failed therapeutic attempt with the R-ICE regimen, a switch to the BV+DHAP protocol was made. A PR was achieved, enabling the patient to proceed to transplantation. Subsequent to AHSCT, a post-transplant PET/CT scan revealed a new mediastinal nodule. Consequently, the patient underwent mediastinal irradiation, followed by polatuzumab vedotine, rituximab plus bendamustine therapy. The patient eventually passed away due to the progression of the underlying disease. Her overall survival spanned 75 months, with post-NHL survival lasting 17 months. The time interval between the diagnosis of cHL and NHL was 58 months.

Clonality tests

In one patient (Case #5), IgHV gene rearrangements were assessed using PCR, and a consistent pattern was observed for both cHL and DLBCL samples. This finding confirmed a common origin and a clonal relationship between these two malignancies.

NGS was conducted in five of the six patients, with one patient not undergoing evaluation. The results of these NGS analyses are summarized in Table 1. Clonal linkage was established in the first patient, although not between the cHL and HG-BCL histological samples. Instead, the clonal linkage was identified between the first lymph node sample with reactive abnormalities and the HG-BCL samples. Additionally, both NOTCH2 and CCND3 gene variants were detected. Remarkably, the presence of HRS cells was also detectable in the revised reactive lymph node. Unfortunately, the second histological sample representing cHL was not available for further molecular analysis. Nevertheless, it is highly likely that a clonal relationship or niche exists between cHL and HG-BCL.

## Discussion

The elevated risk of secondary malignancies among cHL patients is a well-documented phenomenon. A comprehensive study conducted by a Dutch working group involved an overview of nearly four thousand cHL patients who had achieved a minimum of five years of overall survival, spanning the years of 1965 to 2000. The study aimed to investigate the frequency of secondary malignancies and identified 757 cases of solid tumors and 147 cases of hematological malignancies, of which 104 were NHLs. The 30-year cumulative incidence of NHL was calculated to be 3.7 (95% CI 3.0-4.6). Remarkably, when comparing the periods from 1965-1976 to 1989-2000, the risk of developing a secondary NHL, myelodysplastic syndrome (MDS), or leukemia had notably decreased to less than half. Further analysis of treatment modalities revealed that splenectomy, mantle field irradiation, and cumulative doses of procarbazine exceeding 8 mg/m^2^ significantly increased the risk of secondary NHL. Fortunately, these treatment methods are now rarely utilized [10].

In a separate analysis based on SEER (Surveillance, Epidemiology, and End Results) data, U.S. researchers examined the medical records of 26,826 cHL patients treated between 1992 and 2009. They discovered that the cumulative incidence of NHL in this cohort was 2.5% (95% CI: 2.1-2.89). Notably, cases of concomitant cHL-NHL were excluded from this trial [10].

The German Hodgkin Study Group (GHSG) conducted a study focused on the incidence of secondary NHL among patients who participated in the HD7-HD15 clinical trials between 1993 and 2008. Their investigation revealed a 10-year cumulative incidence of NHL to be 1.5% (95% CI: 1.3-1.7). The median time between the initial diagnosis of cHL and the subsequent NHL diagnosis was 44 months, with a range of 21 to 84 months [9].

In the last two studies, no correlation was observed between the type of treatment and the risk of developing secondary NHL. This lack of association is unsurprising as the toxicity of cHL treatments has significantly diminished since the 1990s. Both studies did reveal that sNHL was more prevalent among cHL patients diagnosed at an older age. However, it is worth noting that this age-related increase in incidence was also observed in patients with de novo NHL [9,11].

We found the incidence of secondary NHL among cHL patients to be 3.66%. In our investigation, we deliberately included and examined cases where the diagnosis of NHL was confirmed shortly after the diagnosis of cHL. The revision of the original histological specimens was deemed clinically necessary and justifiable in all such cases. In each instance, an expert hematopathologist confirmed the initial diagnosis of cHL during the revision process.

The exact etiology behind the development of secondary NHL remains elusive, likely stemming from a multifactorial interplay. Factors such as genetics, environmental influences, and immunodeficiency induced by treatment and cHL itself may act as predisposing factors. Notably, the reduction in treatment-related toxicity over the past few decades has diminished its role as a significant provoking factor. However, recent data suggests that immune checkpoint inhibitor treatments, specifically PD-1 inhibitors, can induce clonal evolution in cHL, even after prolonged remission. This effect may be attributed to the PD-1 inhibitor's impact on immunoregulation [9-12].

Aggressive B-cell lymphomas, predominantly DLBCL, are the most common type of secondary NHLs, a pattern also observed among our patients. However, the literature suggests that indolent B-cell and T-cell lymphomas are not uncommon in these cases. It is important to note that the treatability of these secondary lymphomas can be adversely affected by the advanced age of patients and their reduced tolerance for treatment due to prior therapies and the use of cross-resistant modalities [9-11].

Among our patients, we confirmed the clonal relationship in three cases of secondary NHL development (Cases #4, #5, and #6). All three patients were women with varying age ranges, with only one patient being older than 65 years. In one patient, IGHV gene rearrangement analysis established a clonal relationship between the two lymphomas, suggesting that the immunodeficiency induced by cHL and its treatment, as well as specific anti-HL therapy, may lead to clonal selection and the development of DLBCL.

In the case of the older woman, surgical removal of the solitary lesion resulted in long-term disease-free survival. For another patient (Case #4), clonality testing was not performed, but the histological morphology of DLBCL (Figure 2) indicated transformation from cHL. This patient proved to be refractory to conventional chemotherapy regimens but experienced durable remission following treatment with the PD-1 inhibitor nivolumab. After nearly two years of disease-free status, a histopathological examination of nodal relapse confirmed DLBCL. In this patient's case, it is likely that nivolumab-induced clonal evolution occurred, a phenomenon documented in the literature and confirmed by NGS analysis of serial liquid biopsy specimens obtained during the longitudinal follow-up of cHL patients [12,13].

In the case of our other three patients, it is plausible that the two distinct histological types of lymphoma were not formed sequentially but rather were synchronous or discordant at the time of cHL detection. While the coexistence of two different histological types of lymphoma within the same lesion is indeed rare, the presence of distinct histological types of lymphoma at different anatomical sites is possibly more common than reflected in the existing literature. Notably, 12.9% of DLBCL patients have been reported to exhibit concurrent indolent NHL, although the potential for an aggressive transformation of the indolent process is always a concern in such cases [14,15].

The co-occurrence of cHL and NHL is a well-documented phenomenon, as evident from numerous case reports [7,8,14,16]. Composite lymphomas, which manifest within a single lymph node or lesion, are relatively easier to detect through lymph node excision and histopathological review, although such cases are rare. Conversely, synchronous or discordant lymphomas occurring in different anatomical locations can only be identified by obtaining samples from multiple sites. This practice is not particularly common in routine clinical settings, especially when the clinician already has a confirmed diagnosis of a lymphoproliferative disease that aligns with the patient's clinical presentation. This might explain why only a fraction of cases involving multiple lymphomas are detected [14,16].

Upon retrospective analysis, it becomes apparent that synchronous NHL was a possibility in all three of our primary refractory patients. Composit lymphoma was not found in our work, so when we mention synchronous lymphoma, we are talking about cHL and NHL, which appear heterogenically spatial. In the first two patients, the staging PET/CT scans displayed notably higher maximal standard uptake values (SUVmax) in the mediastinal region compared to other involved areas. Meanwhile, the third patient presented with a residual lesion solely in the mediastinum during interim scans, a scenario not uncommon for cHL. However, at the time of their initial diagnosis, thoracic histological sampling was not considered, as the risk of the intervention outweighed the expected benefit at that moment in time, and the histological diagnosis appeared to be consistent with the radiological findings. This highlights the critical importance of effective communication between the clinician and the hematopathologist. In cases where the clinical behavior of the disease deviates from the expected norm, reviewing the histological sample or conducting a rebiopsy is highly recommended.

The development of lymphomas of different histological types occurring simultaneously or sequentially may or may not be indicative of a clonal relationship; they can also arise independently. In our group of patients with relapsed lymphomas that developed sequentially, we had one case where IGHV gene rearrangement and another where morphology supported the notion of a common origin, but this was not confirmed in either case among the primary refractory patients (Cases #1-3). However, the potential for a synchronous dual process in the first patient is bolstered by the fact that the NOTCH2 and CCND3 gene variant, later detected in the HG-BCL, was already present in the initial reactive lymph node that was later histologically revised as cHL.

Various methods are available for confirming clonal linkage. When applicable, characteristic breakpoints can be examined through fluorescence in situ hybridization (FISH), although this is of limited relevance for cHL. However, BCL2 or possibly BCL6 rearrangements may be present in certain cases [14]. DNA can be isolated and analyzed from histological specimens, and the analysis of circulating tumor DNA (ctDNA) in peripheral blood is also a valuable approach. PCR testing of specific sequences of IGH and IGK genes (IGH VH-JH, IGK Vk-Jk, Vk-Kde/intron-Kde) is the most commonly employed method to confirm clonality, and targeted NGS is becoming increasingly available for detecting tumor-specific gene variants or mutations. However, the assessment of clonality in cHL can be challenging due to the fact that in tissue samples, tumor HRS cells constitute only a small fraction, typically around 1-2%, within a mixed cell lymphoid population. This makes it difficult to detect monoclonal immunoglobulin rearrangements or mutations specific to tumor cells. The sensitivity of these assays can be improved through laser microdissection of HRS cells [12,14].

Searching for clonal linkage, we used PCR testing of IGH and targeted NGS testing to detect tumor-specific mutations or gene variants. PCR technique shows a general IgVH rearrangement pattern. That means the general IgVH rearrangement pattern is present in all malignant B-cells and is not dependent on any additional gene mutations. Meanwhile, with the NGS technique, common mutations can be found in tumor cells that gain during oncogenesis. So, clonal linkage can be approved with both methods in a different way. The best scenario to find the most linkage would be for every sample to undergo NGS and IgVH rearrangement PCR testing. Still, unfortunately, the study's retrospective design limits our abilities. Additionally, a clear limitation of our molecular studies was the lack of access to all patients and histological samples in sufficient quantity and quality. However, even with these limitations, we could prove the clonal connection with molecular pathologic methods in two cases. In a retrospective study, it is often not possible to replace these samples. While core biopsies are less invasive for the patient and can expedite the diagnostic process, it is a recognized fact that surgical excision of the lymph node can enhance the accuracy of the diagnosis and facilitate additional investigations, if needed. As our understanding of pathogenesis and prognosis deepens, and diagnostic methods advance, there may be a growing need for the retrospective examination of histological specimens for both clinical and research purposes.

Liquid biopsies involve the analysis of peripheral blood or other bodily fluids, such as cerebrospinal fluid. These non-invasive samples offer several advantages, including reduced stress for the patient and the ability to conduct repeated tests throughout their treatment and follow-up. Liquid biopsies enable the analysis of circulating free DNA (cfDNA) and ctDNA, with methods tailored to the specific diagnostic, prognostic, therapeutic efficacy, or relapse detection needs [12,14,17]. While initially more prevalent in solid tumors, liquid biopsies are increasingly being explored and used in hematological malignancies, including acute myeloid leukemia, multiple myeloma, and lymphomas. In cHL, changes in cfDNA levels during treatment show promise as potential prognostic indicators, though prospective studies are required to confirm this hypothesis [17]. PCR analysis of IGHV gene rearrangements and targeted NGS for somatic mutations in ctDNA can facilitate diagnosis and early relapse detection. The correlation between NGS analysis of tissue samples and corresponding liquid biopsy specimens in lymphomas has proven effective and concordant in detecting somatic mutations. Furthermore, liquid biopsies have identified additional abnormalities in cHL patients, which could aid in subclone identification [17-19]. Standardization of NGS assays for cHL, greater accessibility to laser microdissection, and larger prospective studies are required.

The main limitations of our work were the low quantity of the histological sample in some cases and the low number of patients, as it is an infrequent scenario. In some cases, there were not enough histopathological samples, or the quality of the sample was poor for molecular examinations. As it is a retrospective examination, there was no chance for resampling. 

## Conclusions

In summary, we have observed several forms of cHL and NHL associations among our patients. Unlike other studies, we did not exclude potentially synchronous NHL cases. Reviewing our data, all three synchronous NHLs responded poorly to treatment, post-NHL survival was measured in a few months, whereas in three sequential NHLs, one patient was still alive, one patient succumbed to COVID-19, and the third case had a post-NHL survival of 15 months. Our results suggest that synchronous NHL has a worse prognosis compared to sequential cases. NGS examinations show that in some cases mutations can be identified that accompany the tumor cells throughout their clonal evolution, with additional mutations later on. In the future, NGS-based processing of liquid biopsy samples can overcome the limitations resulting from the spatial heterogeneity of lymphoid malignancies. Over the long term, this identification could lead to early patient selection and alternative treatment strategies, ultimately leading to improved prospects for cure.
